# Phase 1 trial evaluating safety and pharmacokinetics of HIV-1 broadly neutralizing mAbs 10E8VLS and VRC07-523LS

**DOI:** 10.1172/jci.insight.175375

**Published:** 2024-04-08

**Authors:** Seemal F. Awan, Amarendra Pegu, Larisa Strom, Cristina A. Carter, Cynthia S. Hendel, LaSonji A. Holman, Pamela J. Costner, Olga Trofymenko, Renunda Dyer, Ingelise J. Gordon, Ro Shauna S. Rothwell, Somia P. Hickman, Michelle Conan-Cibotti, Nicole A. Doria-Rose, Bob C. Lin, Sarah O’Connell, Sandeep R. Narpala, Cassandra G. Almasri, Cuiping Liu, Sungyoul Ko, Young D. Kwon, Aryan M. Namboodiri, Janardan P. Pandey, Frank J. Arnold, Kevin Carlton, Jason G. Gall, Peter D. Kwong, Edmund V. Capparelli, Robert T. Bailer, Adrian B. McDermott, Grace L. Chen, Richard A. Koup, John R. Mascola, Emily E. Coates, Julie E. Ledgerwood, Martin R. Gaudinski, the VRC 610 study team

**Affiliations:** 1 Vaccine Research Center, National Institute of Allergy and Infectious Diseases, National Institutes of Health, Bethesda, Maryland, USA.; 2 Department of Microbiology and Immunology, Medical University of South Carolina, Charleston, South Carolina, USA.; 3 School of Medicine and Skaggs School of Pharmacy and Pharmaceutical Sciences, University of California San Diego, San Diego, California, USA.; 4 The VRC 610 study team is detailed in Supplemental Acknowledgments.

**Keywords:** AIDS/HIV, Clinical trials, Immunoglobulins, Pharmacology

## Abstract

**BACKGROUND:**

Broadly neutralizing monoclonal antibodies (bNAbs) represent a promising strategy for HIV-1 immunoprophylaxis and treatment. 10E8VLS and VRC07-523LS are bNAbs that target the highly conserved membrane-proximal external region (MPER) and the CD4-binding site of the HIV-1 viral envelope glycoprotein, respectively.

**METHODS:**

In this phase 1, open-label trial, we evaluated the safety and pharmacokinetics of 5 mg/kg 10E8VLS administered alone, or concurrently with 5 mg/kg VRC07-523LS, via s.c. injection to healthy non–HIV-infected individuals.

**RESULTS:**

Eight participants received either 10E8VLS alone (*n* = 6) or 10E8VLS and VRC07-523LS in combination (*n* = 2). Five (*n* = 5 of 8, 62.5%) participants who received 10E8VLS experienced moderate local reactogenicity, and 1 participant (*n* = 1/8, 12.5%) experienced severe local reactogenicity. Further trial enrollment was stopped, and no participant received repeat dosing. All local reactogenicity resolved without sequelae. 10E8VLS retained its neutralizing capacity, and no functional anti-drug antibodies were detected; however, a serum *t_1/2_* of 8.1 days was shorter than expected. Therefore, the trial was voluntarily stopped per sponsor decision (Vaccine Research Center, National Institute of Allergy and Infectious Diseases [NIAID], NIH). Mechanistic studies performed to investigate the underlying reason for the reactogenicity suggest that multiple mechanisms may have contributed, including antibody aggregation and upregulation of local inflammatory markers.

**CONCLUSION:**

10E8VLS resulted in unexpected reactogenicity and a shorter *t_1/2_* in comparison with previously tested bNAbs. These studies may facilitate identification of nonreactogenic second-generation MPER-targeting bNAbs, which could be an effective strategy for HIV-1 immunoprophylaxis and treatment.

**TRIAL REGISTRATION:**

Clinicaltrials.gov, accession no. NCT03565315.

**FUNDING:**

Division of Intramural Research, National Institute of Allergy and Infectious Diseases, NIH.

## Abstract 2

The 10E8VLS bNAbs was found to be safe in a phase 1 trial but is reactogenic and has a shorter half-life compared to previously tested bNAbs.

## Introduction

The HIV/AIDS epidemic has caused profound global morbidity and mortality, with 84.2 million people infected and 40.1 million deaths due to AIDS-related illnesses since 1983 (1). Broadly neutralizing monoclonal antibodies (bNAbs) provide a potential strategy for HIV infection prevention via immunoprophylaxis and a potential strategy for treatment by reducing viremia and limiting drug-resistant viral variants in individuals living with HIV-1 infection (2–5). Additionally, characterization of bNAbs can provide insight into HIV-1 neutralization mechanisms and, thus, inform rationale vaccine design (6).

Each bNAb targets a distinct site of the HIV-1 envelope glycoprotein (Env) structure, such as the CD4-binding site, V1/V2 apex, V3-glycan region, membrane-proximal external region (MPER), and the interface between gp120 and gp41 (7–11). bNAbs have been engineered to extend serum *t_1/2_* and improve pharmacokinetic (PK) profiles, offering the potential for reduced dosing requirements and product cost (12–15). These properties may be particularly advantageous in resource-poor settings where access to antiretroviral therapy (ART) and poor compliance are problematic.

The Vaccine Research Center (VRC) (National Institute of Allergy and Infectious Diseases [NIAID], NIH, Bethesda, Maryland, USA) has developed several bNAbs targeting the CD4 binding site (VRC01, VRC01LS, VRC07-523LS, VRC01.23LS) and has developed a bNAb targeting the MPER region (10E8VLS) (9, 14, 16–22). VRC07-523LS is a clonal relative of VRC01 and was engineered to improve neutralization potency and breadth, as well as *t_1/2_* through the addition of a leucine and serine (LS) mutation (2 amino acid substitution, M428L and N434S), thereby increasing binding affinity to the neonatal Fc receptor (14). VRC07-523LS has been demonstrated to be safe and well tolerated by both i.v. and s.c. administration routes and exhibited superior viral neutralization characteristics compared with previously evaluated bNAbs, including VRC01 and VRC01LS (23).

The Membrane-Proximal External Region (MPER) is a highly conserved region of the Env gp41 subunit near the lipid bilayer viral surface that plays an important role in membrane fusion (24). The 10E8 WT bNAb was isolated from an HIV-1 slow progressor and neutralizes 98% of viruses in a 181-multiclade HIV-1 pseudovirus panel at an inhibitory concentration IC_50_ of < 50 µg/mL (25). Subsequent modifications improved solubility, stability, potency, and *t_1/2_*, resulting in 10E8VLS (26, 27). The 10E8VLS and VRC07-523LS bNAbs target the Env at distinct epitopes: 10E8VLS recognizes the MPER and proximal viral membrane lipid of gp41, while VRC07-523LS recognizes the CD4-binding site of gp120. Moreover, a combination bNAb strategy targeting independent epitopes on the Env may exert additive effects on neutralization activity and reduce the likelihood of viral resistance and escape (28–31).

We report here the safety, PK, and immunogenicity findings of a phase 1, open-label clinical trial evaluating 10E8VLS administered alone, and concurrently with VRC07-523LS, via s.c. injection. This trial resulted in a study termination due to unexpected moderate to severe local site reactogenicity and lower-than-expected human PK data. We found that multiple immune-related mechanisms involving 10E8VLS may have contributed to the observed local site reactogenicity and report the pathophysiologic causes likely leading to the reactogenicity and study termination.

## Results

### Study population.

Enrollment commenced on July 10, 2018, and was closed to accrual on September 21, 2018. Study pause criteria were not met; however, the study was voluntarily closed by the Clinical Trial and IND sponsor (VRC, NIAID, NIH) due to concerns regarding local reactogenicity observed in 6 of 8 participants following administration of 10E8VLS or of 10E8VLS and VRC07-523LS, including 1 Grade 3 injection-site erythema at a 10E8VLS injection site.

**Figure 1:**
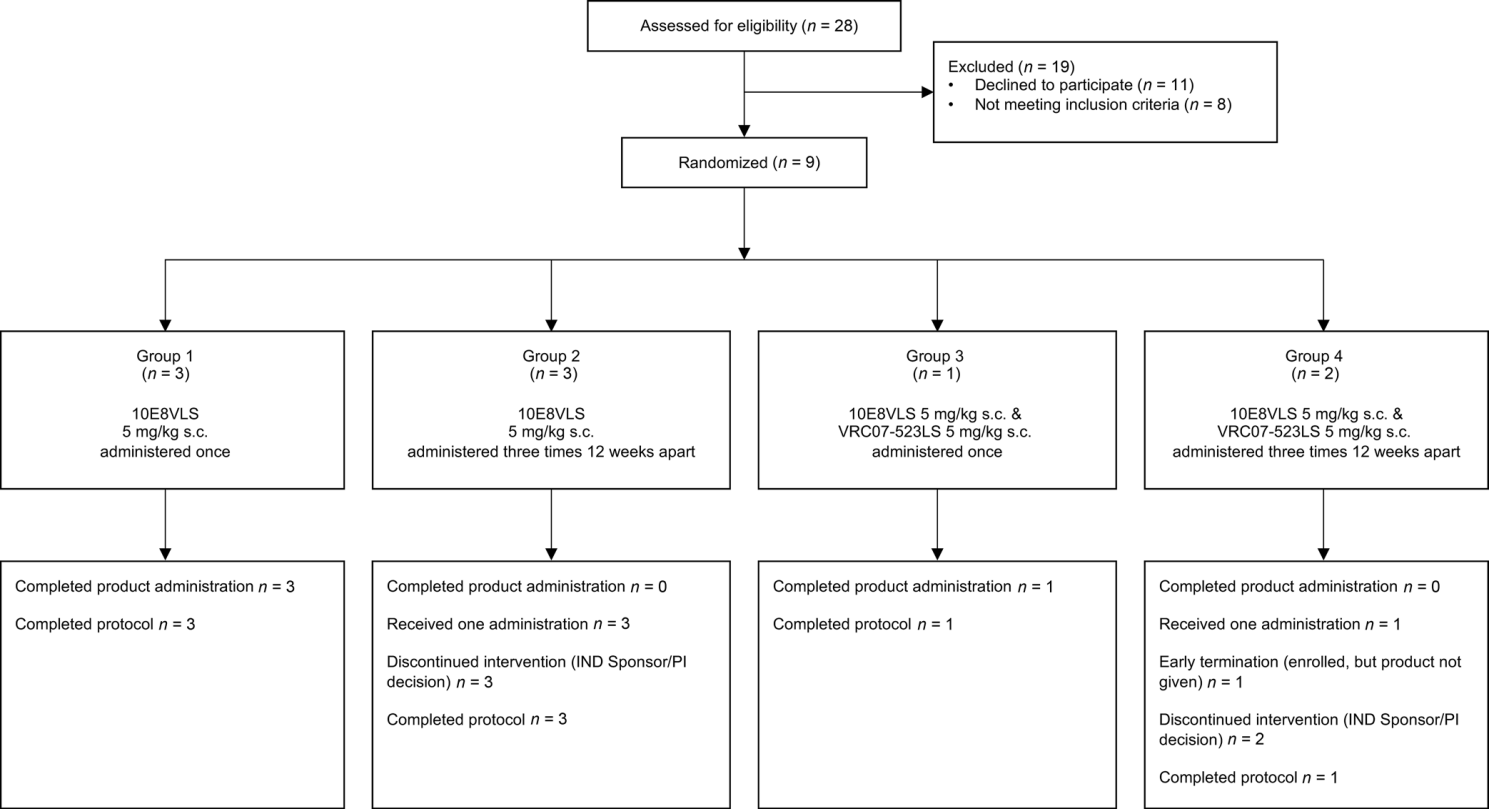
CONSORT diagram. One volunteer in Group 4 enrolled in the study but did not receive 10E8VLS and VRC07-523LS, and all other participants only received 1 administration of 10E8VLS and/or VRC07-523LS due to study termination.

Nine participants were enrolled in the trial, including 6 males (66.7%) and 3 females (33.3%) (Table 1). Six participants received a single administration of 5 mg/kg s.c. 10E8VLS only, and 2 participants received administrations of both 5 mg/kg s.c. 10E8VLS and 5 mg/kg s.c. VRC07-523LS in separate sites of the abdomen on the day of administration (Figure 1). One participant in the combined 10E8VLS and VRC07-523LS group did not receive any products, and every participant in repeat-dosing groups did not receive additional product doses due to the study termination (Figure 1).

### Safety.

**Figure 2:**
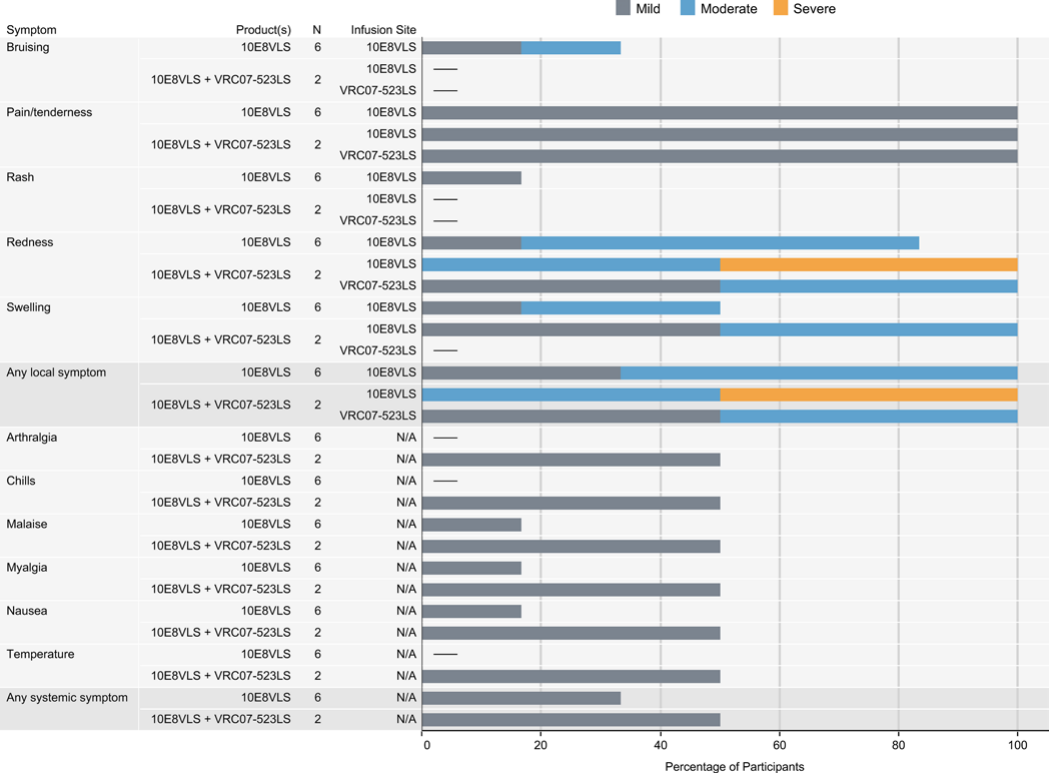
Maximum solicited local and systemic reactogenicity following administration. 10E8VLS and VRC07-523LS were administered as separate injections at different sites on the same day and, therefore, have distinct local reactogenicity reported for each product.

Product safety and tolerability were the primary objectives and are reported herein. Solicited local reactogenicity was mild to severe and resolved without sequelae (Figure 2 and Supplemental Tables 1 and 2; supplemental material available online with this article; https://doi.org/10.1172/jci.insight.175375DS1). Of the participants who received 1 s.c. administration of 5 mg/kg of 10E8VLS alone, all 6 participants (100%) reported mild pain/tenderness; 2 participants (33%) reported mild or moderate bruising; 5 participants (83%) reported mild or moderate redness; 3 participants (50%) reported mild or moderate swelling; and 1 participant (17%) reported mild pruritus.

**Figure 3:**
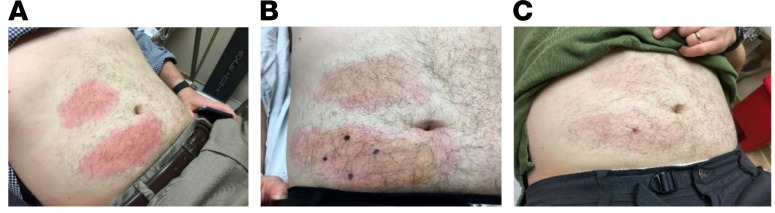
Local reactogenicity experienced by participant 7 after product administration. (**A**) Day 2 after product administration. RLQ reactogenicity quantified as 17 × 8 cm. RUQ reactogenicity quantified as 11 × 6.5 cm. (**B**) Day 3 after product administration. RLQ: 18 × 7 cm. RUQ 13 × 7 cm. (**C**) Day 7 after product administration. RLQ: 16 × 6 cm. RUQ 10 × 5 cm. VRC07-523LS was administered on the left side of the abdomen.

Two participants received 5 mg/kg each of 10E8VLS and VRC07-523LS. Participant 8 experienced mild pain/tenderness, mild swelling, and moderate redness at the 10E8VLS injection site as well as mild redness at the VRC07-523LS injection site. Participant 7 had notable local reactogenicity, which included mild pain/tenderness, moderate swelling, and severe redness at the 10E8VLS injection site (Figure 3 and Supplemental Table 1) as well as moderate redness at the VRC07-523LS injection site. The 10E8VLS volume was administered as 2 separate s.c. injections in the right lower quadrant (RLQ) and in the right upper quadrant (RUQ) of the abdomen. Injection-site redness experienced by participant 7 measured 12 × 5 cm and 9.5 × 5 cm in the RLQ and RUQ, respectively, on day 1 and increased to a maximum of 18 × 7 cm and 13 × 7 cm, respectively, on day 3 (Figure 3B). A skin biopsy was performed and reviewed by the dermatopathology team at the NIH Clinical Center (Bethesda, MD, USA). This skin biopsy was obtained on day 3 and showed superficial and deep perivascular chronic inflammation with panniculitis and eosinophils, consistent with large injection-site reaction without vasculitis (Supplemental Figure 5 and Supplemental Methods). The redness from 10E8VLS resolved in approximately 10 weeks (71 days). The VRC07-523LS volume was administered as 2 separate s.c. injections in the left lower quadrant (LLQ) and in the left upper quadrant (LUQ) of the abdomen. Injection site redness in participant 7 measured 4.5 × 4.5 cm in the LUQ and 5.5 × 3.3 cm in the LLQ. The redness from VRC07-523LS initially resolved after 1 day but then recurred 12 days later and lasted for 46 days. The participant was assessed weekly, and this adverse event (AE) resolved 46 days after onset with no additional symptoms. This redness was the only AE that occurred during the trial that was determined to be related to product administration.

In summary, among 6 participants who received only 10E8VLS, 2 (33.3%) reported mild symptoms and 4 (66.7%) reported moderate symptoms at the injection site (Figure 2). Among 2 participants who received both 10E8VLS and VRC07-523LS, 1 (50%) reported moderate symptoms and 1 (50%) reported severe symptoms (redness) at the 10E8VLS injection site; furthermore, 1 (50%) reported mild symptoms and 1 (50%) reported moderate symptoms at the VRC07-523LS injection site.

Systemic reactogenicity was absent or mild in severity when reported and resolved without sequelae (Figure 2 and Supplemental Tables 1 and 2). Among 6 participants who received 10E8VLS alone, 4 (66.7%) had no systemic symptoms, while 1 (16.7%) reported mild myalgia and 1 (16.7%) reported mild malaise and nausea. One (50%) participant who received both 10E8VLS and VRC07-523LS reported mild systemic symptoms (malaise, myalgia, chills, nausea, fever, joint pain) on day 1; these symptoms resolved the same day with ibuprofen.

### PK.

**Figure 4:**
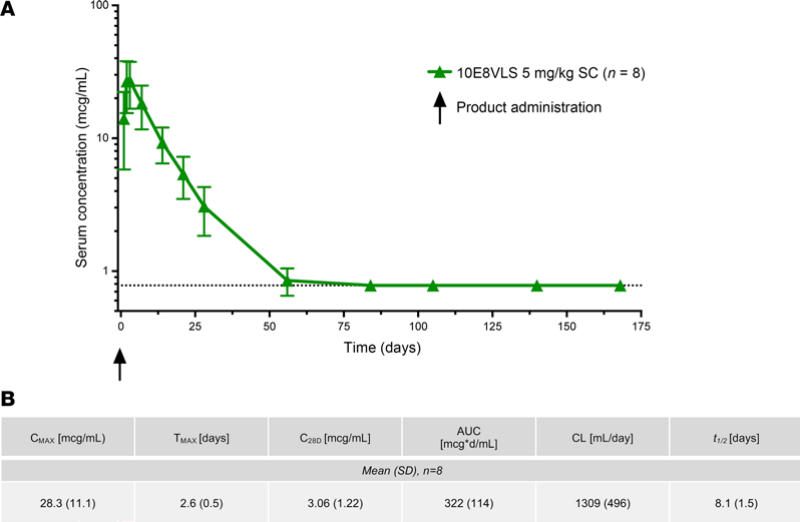
Pharmacokinetics of 10E8VLS. (**A**) Mean antibody serum concentration and SD (µg/mL) of 10E8VLS administered at 5 mg/kg s.c. (**B**) PK parameters. All patients are included who received 10E8VLS (Group 1, 2, 3, and 4; *n* = 8) alone or in conjunction with VRC07-523LS. VRC07-523LS serum concentrations are depicted in Supplemental Figure 1. The black dotted line at *y* = 0.78 indicates the limit of detection. C_max_, maximum serum concentration; T_max_, time to maximum serum concentration; C_28D_, Day 28 serum concentration; CL, clearance.

Evaluation of antibody PK was the trial’s secondary objective ,and quantification of serum concentrations was performed for all recipients of 10E8VLS and VRC07-523LS. Analysis of the PK parameters was performed for all participants who received 10E8VLS (Figure 4). Mean (±SD) maximum serum concentration (C_max_) value following a single administration of 10E8VLS at 5 mg/kg s.c. was 28.3 ± 11.1 µg/mL. The mean (±SD) time to maximum serum concentration (T_max_) was 2.6 (0.5) days. 10E8VLS concentration fell rapidly following peak concentration, with a mean (±SD) serum elimination *t_1/2_* of 8.1 ± 1.5 days and a clearance rate (CL) of 1,309 ± 496 mL/day. The PK profile of VRC07-523LS (*n* = 2) was consistent with previously reported parameters (23). At Day 28, the mean (±SD) concentration (C_28D_) of 10E8VLS was 3.1 ± 1.2 µg/mL, less than half of VRC07-523LS (Supplemental Figure 1). 10E8VLS concentrations were below the assay limit (<0.8 µg/mL) at week 8 in 7 of 8 participants, while both VRC07-523LS profiles were > 1 µg/mL at Day 140 (Supplemental Figure 1).

### Neutralization potency and breadth.

**Figure 5:**
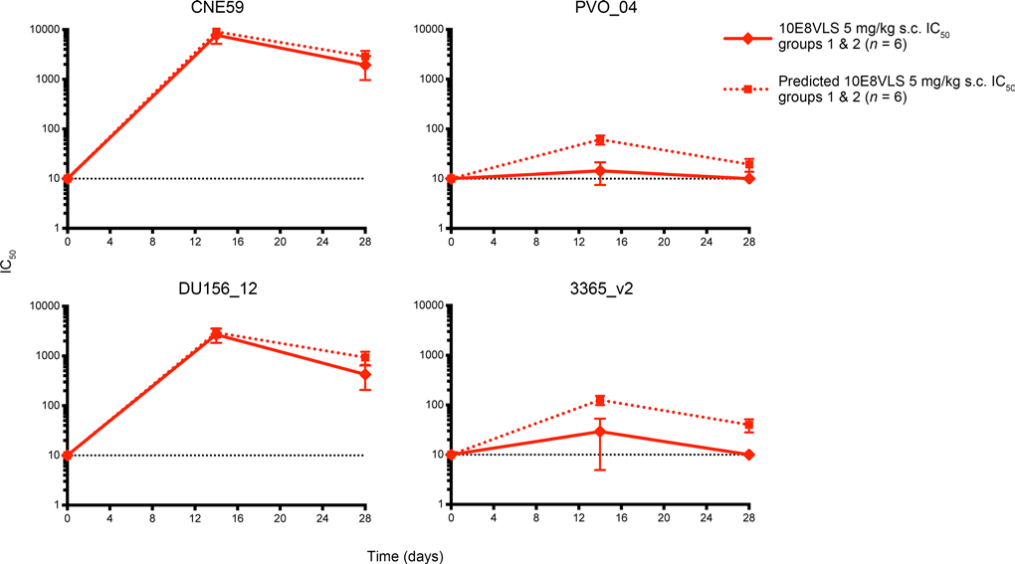
Neutralization characteristics of sera containing 10E8VLS. Mean ± SD of experimental vs predicted IC_50_ neutralization titers of 10E8VLS in Group 1 and 2 patients (*n* = 6) against genetically diverse HIV-1 Env–pseudoviruses. The black dotted line at *y* = 10 indicates the assay’s limit of detection. Experimental values were generated by HIV-1 pseudovirus neutralization assay, and predicated values were calculated as 10E8VLS serum concentration/control 10E8VLS IC_50_.

As an exploratory objective, we tested sera from participants who received 5 mg/kg 10E8VLS alone against a panel of 6 genetically diverse HIV-1 Env–pseudoviruses out to 28 days after administration to assess if the infused antibody retained its neutralization characteristics in serum. When comparing predicted versus experimental neutralization characteristics, 10E8VLS neutralized as expected for CNE59 (clade AE) and DU156_12 (clade C) but slightly lower than predicted for PVO_04 (clade B) and 3365_v2 (clade A). However, these differences are within the expected variability of the assay and are not interpreted as biologically meaningful. Pseudoviruses not shown, SIV_MAC251.30 and T266-60 (clade AG), represent the negative assay control and a 10E8VLS-resistant virus (Figure 5).

### Anti-drug antibody detection.

Participants were tested for anti-drug antibody (ADA) development following 10E8VLS and VRC07-523LS administration (Supplemental Table 3). Functional ADA was not detected in any sample after administration of 10E8VLS or VRC07-523LS.

### Participant IgG1 genetic marker allotype analysis.

Analysis of the IgG1 genetic marker (GM) allotypes of all enrolled participants (*n* = 9) for possible influence on PK was performed as an exploratory trial analysis. These antigenic determinants are located on IgG heavy chains and can affect the PK of antibodies through differing FcRn affinities (32). No marked allotypic effects on the PK parameters were observed following 10E8VLS administration in our small study population.

### Mechanistic investigation of observed reactogenicity.

Mechanistic investigations were performed to seek a cause of the unexplained reactogenicity and shorter than expected *t_1/2_*. These included adipocyte binding and activation, product aggregation and complement activation, and activation of monocytes in the presence of 10E8VLS.

### Binding and activation of primary preadipocytes and adipocytes.

**Figure 6:**
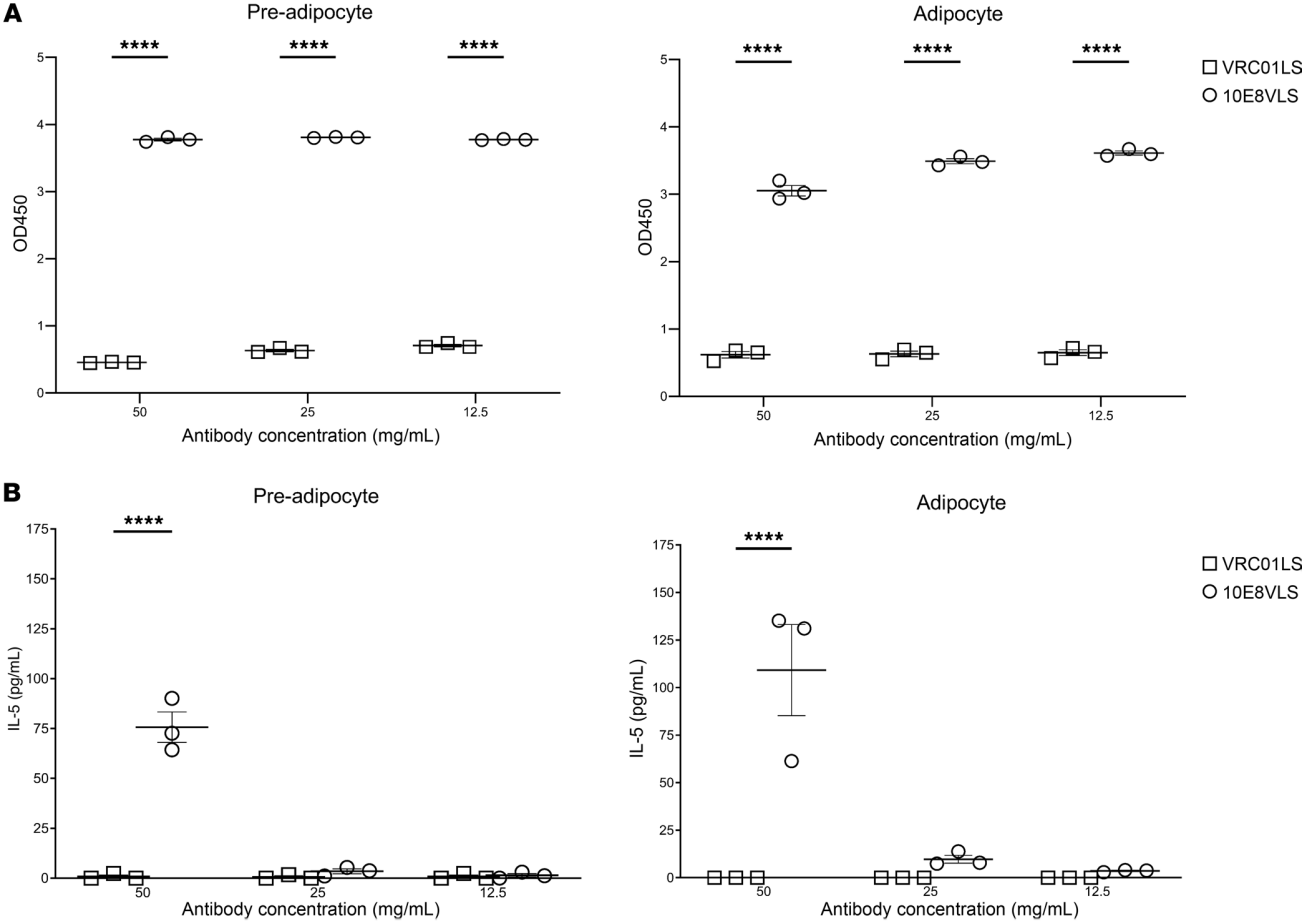
Binding to preadipocytes and adipocytes by 10E8VLS and VRC01LS followed by IL-5 secretion. (**A**) Binding of 10E8VLS or VRC01LS to primary human preadipocytes — or in vitro–differentiated adipocytes derived from 3 healthy donors at concentrations of 50, 25, and 12.5 mg/mL — was detected using an HRP-conjugated secondary antibody. The OD450 values indicative of binding are shown for each antibody concentration tested for each donor; the bar denotes the mean, with the error bars representing the SEM. (**B**) Primary human preadipocytes and adipocytes from 3 healthy donors were incubated with 50, 25, and 12.5 mg/mL 10E8VLS or VRC01LS. Culture supernatant was taken after a 4.5-hour incubation and assayed for secreted cytokine levels by a multiplex assay. The IL-5 levels are shown for each antibody concentration tested for each donor; the bar denotes the mean, with the error bars representing the SEM. For statistical analysis, a 2-way ANOVA using the Šidák multiple-comparison test was performed. ****P < 0.0001.

The hallmark of panniculitis is inflammation in the adipose tissue in the hypodermis. We thus attempted to examine the effects of 10E8VLS on human adipose cells, including preadipocytes and adipocytes derived from 3 different lipectomy donors. Both primary human preadipocytes and in vitro differentiated adipocytes showed binding to 10E8VLS but not VRC01LS after incubation with either 50, 25, or 12.5 mg/mL of each antibody (Figure 6A). Antibody concentration was selected to mimic local concentration at the injection site during the time of local reactogenicity symptom onset. VRC01LS was used as a control, as it has not caused local reactogenicity in previous clinical trials (17). To further investigate, we mapped the cytokines produced by primary human preadipocytes and adipocytes in the presence of 10E8VLS or VRC01LS at 12.5, 25, and 50 mg/mL to mimic the site of s.c. injection (Figure 6A and Supplemental Figure 2). Preadipocytes and adipocytes derived from all 3 donors released low levels of IL-5 after a 4.5-, 8.5-, or 25-hour incubation with 50 mg/mL 10E8VLS (Figure 6B), whereas neither cell type produced any IL-5 after incubation with VRC01LS.

### Aggregation in presence of serum.

**Figure 7:**
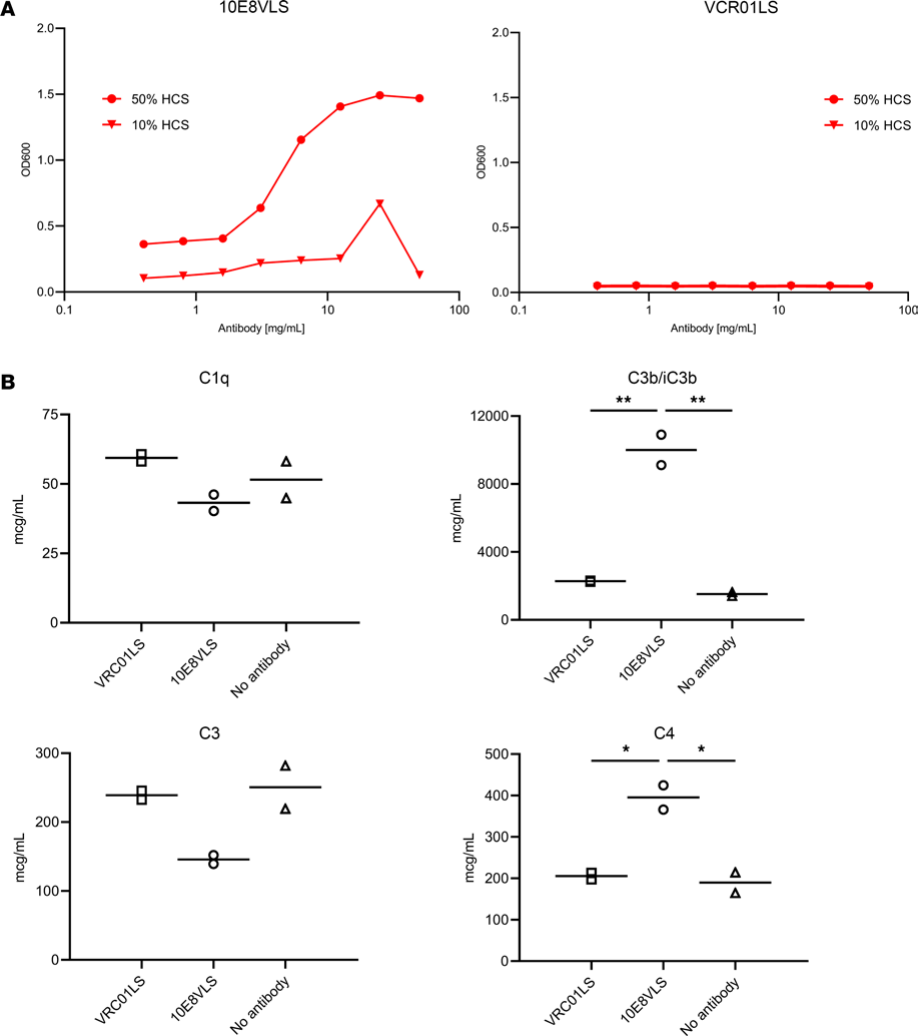
Serum aggregation and activation of complement proteins by 10E8VLS or VRC01LS. (**A**) 10E8VLS or VRC01LS at different concentrations were incubated with either 10% or 50% HCS, and protein aggregation was measured by assessing OD at 600 nm. (**B**) 10E8VLS or VRC01LS at a concentration of 5 mg/mL, or no antibody control (PBS), were incubated with 20% HCS. The activation of complement was measured after a 30-minute incubation by quantifying levels of key complement proteins by a multiplex assay. The levels of C1q, C3, C3b/iC3b, and C4 are shown; the bar denotes the mean from 2 technical replicates. For statistical analysis, 1-way ANOVA using Tukey’s multiple-comparison test was performed. *P < 0.05 and **P < 0.01.

mAbs can interact nonspecifically with diverse molecules including those found in serum when infused at high concentrations (33). Therefore, we assessed the aggregation propensity of 10E8VLS and VRC01LS in the presence of serum. Aggregation of 10E8VLS occurred in medium supplemented with both 10% and 50% human complement serum (HCS) but was not observed for VRC01LS at either HCS dilution (Figure 7A). 10E8VLS aggregation was greater at the higher (50%) HCS dilution and appeared dependent on the concentration of the antibody. No aggregation was observed for VRC01LS in the presence of HCS. Overall, these results suggest that 10E8VLS is prone to aggregation in the presence of serum.

### Activation of complement pathways.

Since aggregated antibodies may lead to activation of complement pathways, we next assessed the effect of 10E8VLS or VRC01LS on complement proteins present in serum. We measured levels of different complement proteins after incubation of 10E8VLS or VRC01LS with human serum. The results indicate that C1q and C3 levels decreased in the presence of 10E8VLS compared with VRC01LS or no antibody control (Figure 7B). Since these proteins are key activators of the classical and lectin complement pathways, their decrease indicates complement activation. Levels of C3b and C4, which are also key mediators of the complement pathway, increased significantly in the presence of 10E8VLS compared with VRC01LS or no antibody controls (Figure 7B). In contrast, the levels of Factor B, a key activator of the alternative complement pathway, did not vary significantly in the presence of either 10E8VLS, VRC01LS, or the no-antibody control, indicating that the alternative complement pathway is not activated by 10E8VLS or VRC01LS (Supplemental Figure 3).

### Activation of monocytes.

**Figure 8:**
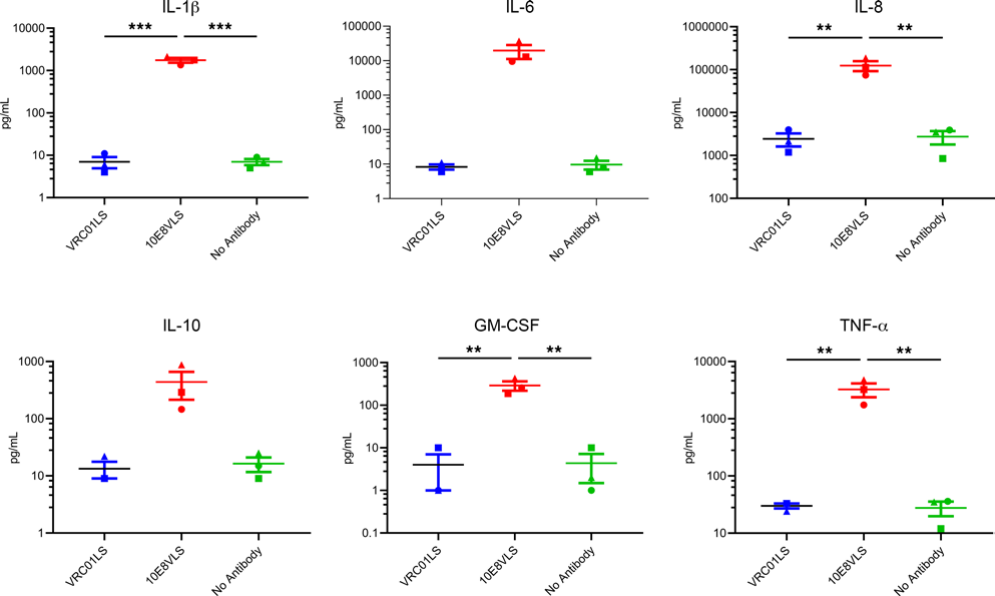
Monocyte activation by 10E8VLS or VRC01LS. Monocytes derived from the peripheral blood from 3 healthy donors were incubated with 100 µg/mL of 10E8VLS, VRC01LS, or no antibody (PBS) for 3 days. The levels of different cytokines in the culture supernatants were assessed by a multiplex assay. The levels of IL-1β, IL-6, IL-8, IL-10, GM-CSF, and TNF-α are shown for each donor; the bar denotes the mean, with the error bars representing the SEM. For statistical analysis, 1-way ANOVA using Tukey’s multiple-comparison test was performed. **P < 0.01 and ***P < 0.005.

PBMCs can be activated by antibody aggregates and lead to production of inflammatory cytokines. We therefore investigated the effect of 10E8VLS or VRC01LS on activation of PBMCs. We found that PBMCs incubated with 10E8VLS, especially ones that had higher proportions of monocytes, secreted inflammatory cytokines like IL-6 and IL-8 (Supplemental Figure 4). We therefore enriched for monocytes and incubated them in the presence of 10E8VLS or VRC01LS. We found high levels of inflammatory cytokines IL-1, IL-6, IL-8, and IL-10, GM-CSF, and TNF-α in the presence of 10E8VLS compared with low or undetectable levels in the presence of VRC01LS or no antibody (Figure 8). These results suggest that 10E8VLS can stimulate monocytes to secrete inflammatory cytokines.

## Discussion

In this small first-in-human phase 1 clinical trial, we found that administration of the HIV-1 bNAb 10E8VLS was safe, although its s.c. administration resulted in unanticipated moderate to severe local reactogenicity. The 8-day *t_1/2_* of 10E8VLS after a single administration at 5 mg/kg s.c. was shorter than expected in comparison with other antibodies with similar LS mutations (26). With the same dosing and administration route, VRC07-523LS has a *t_1/2_* of 36 days and VRC01LS has a *t_1/2_* of 66 days (17, 23). Despite the shorter-than-expected *t_1/2_* of 10E8VLS, it still retained its neutralization characteristics in the plasma after infusion. Additionally, ADA was not detected in any of the individuals infused with 10E8VLS and, therefore, does not explain the short *t_1/2_*.

Due to the unanticipated reactogenicity and shorter-than-anticipated *t_1/2_* for 10E8VLS, the trial was prematurely closed to further product administrations and participant accrual per sponsor (VRC, NIAID, NIH) decision, although the study did not meet pause criteria. This reactogenicity was further investigated, particularly for the 1 participant (participant 7) who received both 10E8VLS and VRC07-523LS and experienced moderate redness at the VRC07-523LS site that lasted 46 days and severe grade 3 redness at the 10E8VLS site that lasted for 71 days. This participant had a history of rosacea that was treated with daily doxycycline. Thus, it is possible that specific host factors related to epidermal function, or perhaps increased skin sensitivity caused by the antibiotic, contributed to severe local redness and length to resolution.

To understand the mechanism underlying the injection-site reactions, a skin biopsy of the Grade 3 10E8VLS injection site redness of participant 7 was performed. The skin biopsy showed lymphocytic inflammation with panniculitis and eosinophil infiltration. Since panniculitis is associated with inflammation in the adipose tissue (34), we assessed the binding and activation of primary preadipocytes and adipocytes with 10E8VLS. We found that 10E8VLS, but not VRC01LS, bound to these adipose cells. VRC01LS was used as a control, since it has not caused this degree of injection site erythema in previous clinical trials (17). In addition, these adipocytes secreted IL-5 after incubation with 10E8VLS. Since IL-5 is a key regulator of eosinophil activation, this may explain the presence of eosinophils seen in the skin biopsy (35, 36).

Additionally, 10E8VLS was noted to form aggregates at high concentrations in the presence of serum. This further prompted an exploration of whether these aggregates can activate the complement system. The complement system is an important component of the innate immune system that provides a first line of defense against pathogens and is also involved in immunological and inflammatory responses (37). There are 3 main pathways (classical, alternative, and lectin) that lead to complement activation; activation is dependent on either the presence of antibody-antigen immune complexes or pathogenic surfaces or components. Activation of the complement system by 10E8VLS was shown by a decrease of complement components C1q and C3 and an increase in the components C3b and C4. These data suggest that 10E8VLS can lead to activation of multiple complement pathways. Therefore, activation of complement by 10E8VLS may have also played a role in the increased local site reactogenicity observed after 10E8VLS administration to humans.

Immediate local injection site reactions have been observed for other therapeutic mAbs and can be explained by various mechanisms that include release of proinflammatory cytokines by immune cells (38). Therefore, we evaluated and compared the ability of 10E8VLS and VRC01LS (control antibody) to induce cytokine release by primary immune cells, particularly human monocytes. We found high levels of inflammatory cytokines secreted by immune cells in the presence of 10E8VLS compared with low or undetectable levels in the presence of VRC01LS or no antibody. These results suggest that 10E8VLS can stimulate monocytes to secrete inflammatory cytokines, and this may have also contributed to the local injection-site reactogenicity after 10E8VLS administration. Our studies assessing the activation of adipocytes, aggregation in serum, complement activation, and monocyte activation by 10E8VLS suggest that multiple immune-mediated mechanisms may have resulted in the observed local reactogenicity. These assays may allow screening of antibody variants for future trials using 10E8VLS derivatives and other bNAbs products.

The small number of participants in this study, while common in phase 1 clinical trials, is a limitation in our study. Due to the small sample size, the conclusions we can draw from our findings are limited and primarily observational. Additional limitations include the decision to discontinue enrollment, product administrations, and sample collections. Furthermore, skin biopsies from multiple participants would have provided additional data to support our findings.

Based on the findings from our mechanistic studies, screening for specific host factor interactions during bNAb development could aid in the development of bNAbs that do not produce the local reactogenicity seen with 10E8VLS. Currently, there are bispecific and trispecific antibodies containing 10E8-related variants in clinical investigation (NCT03875209, NCT03705169). One of these variants, 10E8v4 (ref. 27), which is part of the trispecific antibody under clinical investigation, is missing 2 mutations in the heavy chain (V5R, S100cF) that are present in 10E8VLS, suggesting that these mutations may be associated with the observed local site reactogenicity for 10E8VLS. Therefore these 10E8 variants that contain distinct mutations from those contained in 10E8VLS may be future clinical candidates. A combination bNAb treatment targeting the MPER region in conjunction with another envelope binding site remains an attractive strategy for HIV-1 immunoprophylaxis and treatment.

## Methods

Supplemental Methods are available online with this article.

### Sex as a biological variable

Males and females participated in the clinical trial; sex was not considered as a biological variable.

### Study design

This study was designed as a phase 1, open-label study examining the safety, tolerability, and PK of a single- or repeat-dose regimen of 10E8VLS administered alone or concurrently with VRC07-523LS, via s.c. injection in healthy adults ages 18–60 (ClinicalTrials.gov, NCT03565315). Exclusion criteria included previous receipt of a licensed or investigational mAb, body weight greater than 115 kg, history of severe allergic reaction likely to recur on study, poorly controlled hypertension, receipt of any investigational study agent within 28 days prior to enrollment, or any chronic or clinically significant medical condition. The study was conducted at the NIH Clinical Center by the VRC Clinical Trials Program, and recruitment occurred in the Washington, D.C., USA, and Maryland, USA, area.

### Protocol defined trial objectives

The primary trial objectives were evaluation of the safety and tolerability of a 5 mg/kg dose of 10E8VLS administered alone or concurrently with a 5 mg/kg dose of VRC07-523LS by s.c. injection once or 3 times by repeat-dosing every 12 weeks. The secondary trial objective included evaluation of the PK parameters of 10E8VLS alone or with VRC07-523LS through 24 weeks for both single and repeat-dosing groups. Exploratory trial objectives included evaluation for ADAs in all dosing groups, assessing the neutralization potential of 10E8VLS in participant sera collected through the study, and assessment of participant allotypes for evaluation of allotype-specific effects on PK.

### Study products

Human monoclonal antibodies 10E8VLS (VRC-HIVMAB095-00-AB) and VRC07-523LS (VRC-HIVMAB075-00-AB) were separately produced in stable transfected CHO DG44 cell lines developed by the VRC (NIAID, NIH) and manufactured under current Good Manufacturing Practices (cGMP) at the VRC Pilot Plant operated under contract by the Vaccine Clinical Materials Program (VCMP; Leidos Biomedical Research Inc.). VRC07-523LS is an engineered clonal relative of VRC01 with modifications to improve neutralization breath potency, manufacturability, and extend serum *t_1/2_* through incorporation of the LS mutation in the Fc constant region (13, 14, 23). The 10E8 WT bNAb was isolated at the NIH from an HIV-1 slow progressor (25). Subsequent modifications were made to the WT bNAb to maximize its molecular solubility, stability, potency, and serum *t_1/2_*, thereby producing 10E8VLS (13, 39).

All administration doses were prepared individually by an NIH Clinical Center pharmacist. 10E8VLS was filled at a concentration of 100 ± 10 mg/mL in a sterile, aqueous, buffered solution of 5.25 mL in 10 mL glass vials. VRC07-523LS was supplied at a concentration of 100 ± 10 mg/mL in an isotonic, sterile solution of 6.25 ± 0.01 mL in a 10 mL glass vial. All doses were administered by s.c. injection using needle and syringe to the abdomen, arms, and thighs. Given the weight criterion in this study, the maximum volume needed to administer a 5 mg/kg s.c. dose per bNAb did not exceed 5.75 mL.

### Study procedures

The 5 mg/kg s.c. bNAb dose and regimens were selected based on prior clinical experience with other HIV-1 bNAbs (17, 18, 40). The 4 dosing regimens included 5 mg/kg s.c. 10E8VLS dosed once (Group 1) or 3 times with 12 weeks between each injection (Group 2), or concurrent 5 mg/kg s.c. 10E8VLS and 5 mg/kg s.c. VRC07-5253LS dosed once (Group 3) or 3 times with 12 weeks between each set of injections (Group 4) (Figure 1). The groups ith 10E8VLS 5 mg/kg s.c. and 10E8VLS 5 mg/kg s.c. dosed 3 times were enrolled first, with an interim safety review occurring prior to enrollment of Groups 3 and 4. The first product administration in each group was followed by a 3-day wait period prior to additional product administrations in that group.

All product administrations were monitored by a study clinician. Safety laboratory tests were obtained prior to administration and throughout the study. Participants recorded solicited symptoms for 3 days after each administration, and a clinician assessed the local site on the day of administration, the following day, and 1 week afterward. All AEs were recorded for 56 days after administration, while serious AEs (SAEs) and new chronic medical conditions were recorded throughout the study. AEs were recorded using the Medical Dictionary for Regulatory Activities (MedDRA), and severity was graded using Version 2.0 of the DAIDS Table for Grading the Severity of Adult and Pediatric Adverse Events.

### PK analysis

Independent quantification of 10E8VLS or VRC07-532LS concentration was performed with their respective anti-idiotype as capture antibody (see Supplemental Methods) using electrochemiluminescence immunoassay (ECLIA) on the Meso Scale Discovery (MSD) platform and is described in detail in the Supplemental Methods. A noncompartmental PK analysis was performed on the 10E8VLS concentration data to determine AUC, C_max_, T_max_, concentration at key time points, CL, and elimination *t_1/2_*. Both compartmental and noncompartmental PK analyses were performed on the 10E8VLS concentration data. The noncompartmental analysis was performed using SAS (ver 9.4) to determine AUC, C_max_, T_max_, and concentration at key time points. The compartmental analysis was performed using Nonlinear Mixed Effects Modeling (NONMEM) (ver 7.3) to determine apparent clearance (CL/bioavailability [F]), and the elimination *t_1/2_*.

### HIV pseudovirus neutralization

An HIV pseudovirus neutralization assay was performed with participant sera as previously reported (18). Briefly, sera were evaluated to determine the capacity to prevent the infection of TZM-bl cells (NIH AIDS Research and Reference Reagent Program) by replication-incompetent pseudotyped viruses expressing envelope antigen and the luciferase reporter gene. Neutralization activity was quantitated on a Molecular Devices Paradigm luminometer (Perkin Elmer) by the relative decrease in the luciferase activity as compared with infection of TZM-bl cells in the absence of participant sera. The IC_50_ titer was determined to be the serum dilution that can be interpolated to have 50% of the maximum luciferase activity, as determined by the assay run in the absence of patient sera, using a 5-point parametric curve fitting. A panel of 6 viruses was tested for neutralizing activity spanning a known range of sensitivity for 10E8VLS as well as negative controls. The tested viruses included 3365_v2 (clade A), CNE59 (clade AE), DU156_12 (clade C), PVO_04 (clade B), T266-60 (clade AG), and SIV_MAC251.30 (negative control).

### ADA analysis

A 3-tier assay was used to screen, confirm, and functionally characterize for ADA in the clinical serum samples using the MSD electrochemiluminescence bridging assay in accordance with FDA guidance and as previously reported (23).

### Participant IgG1 GM allotype analysis

IgG1 allotypes GM 3 and GM 17 were genotyped by a TaqMan genotyping assay from Applied Biosystems Inc. as previously described (41). Allotype GM 1 was typed serologically by a standard hemagglutination-inhibition assay (42).

### Mechanistic investigation of observed reactogenicity

#### Antibody production.

VRC01LS was produced in a stable transfected CHO DG44 cell line developed by the VRC (NIAID, NIH) and was manufactured using a process representative of the clinical product at the VRC Pilot Plant operated under contract by the VCMP (Leidos Biomedical Research Inc.).

### Culture of primary human preadipocytes

Primary human preadipocytes from healthy donors who underwent liposuction were cultured following the protocol from the vendor (ATCC). Briefly, the cells were grown in fibroblast basal medium supplemented with 5 ng/mL rhesus FGF-β, 7.5 mM L-glutamine, 50 µg/mL ascorbic acid, 1 µg/mL hydrocortisone hemisuccinate, 5 µg/mL rhesus insulin, and 2% FBS (all from ATCC). The cells were cultured in a tissue culture incubator at 37°C with 5% CO_2_. The medium was replaced every day.

### Differentiation of primary human preadipocytes into adipocytes

Primary human preadipocytes with no more than 4 passages were split in 6-well plates at a density of ~18,000 cells/cm^2^ (43, 44). The cells were cultured in each well with 2 mL preadipocyte growth medium as described above, at 37°C with 5% CO_2_ for 2–3 days. The culture medium was removed, and the monolayer cells were rinsed with PBS. Adipocyte differentiation was initiated upon the addition of 2 mL of prewarmed (37°C) Adipocyte Differentiation Initiation Medium (ADIM; ATCC) and grown at 37°C with 5% CO_2_ for 48 hours. The cells were fed with 2 mL of prewarmed (37°C) ADIM following the removal of half volume of the medium. The cells were maintained for another 48 hours. After 2 mL of medium from each well was discarded, 2 mL of prewarmed Adipocyte Differentiation Maintenance Medium (ADMM; ATCC) was added to each well of cells and the dose was repeated every 48 hours.

### Antibody binding of primary human preadipocytes and adipocytes

Primary human preadipocytes were seeded at a density of ~18,000 cells/cm^2^ in 6-well culture plates. The cells were grown in primary preadipocyte growth medium (ATCC) for 2 additional days, and the medium was changed every day. Adipocytes were prepared as described above. The preadipocytes and corresponding differentiated adipocytes on day 12 after differentiation initiation were incubated in individual growth medium supplemented with 50, 25, or 12.5 mg/mL 10E8VLS or VRC01LS at 37°C for 1 hour. Control cell cultures were supplemented with equal volumes of the formulation buffer for either 10E8VLS (10 mM acetate [J.T. Baker]/phosphate [Fisher], 50 mM NaCl [Alfa Aesar], 100 mM arginine [Acros Organics], 5% sucrose [Sigma-Aldrich], and 0.05% Pluronic F68 [poloxamer 188; Spectrum], pH 6.75) or VRC01LS (25 mM sodium citrate [Macron Chemicals], 50 mM NaCl, and 150 mM L-arginine hydrochloride [J.T. Baker], pH 5.8). After the removal of the culture supernatant, the monolayer cells were washed with 5 mL of sterile PBS solution 3 times. The PBS solution was discarded, and the cells in each well were incubated with 0.75 mL of HRP-conjugated anti–human IgG (Cytiva life sciences, catalog number NA933, 1/500 (V/V)) at 37°C for 30 minutes. The supernatant was then removed, and the cell monolayer was washed with 5 mL of PBS 3 times. To each well of cells after the removal of PBS, 0.75 mL of SureBlue TMB substrate (SeraCare) was added and incubated for 10 minutes. The supernatant was transferred to a 96-well plate, and serial 2-fold dilutions were prepared in PBS. Equal volume of 1N H_2_SO_4_ solution was added to each well to stop the reaction. The absorbance was measured at 450 nm using a SpectraMax Plus 384 Microplate Reader (Molecular Devices).

### Cytokine production by primary human preadipocytes and adipocytes

Primary human preadipocytes and adipocytes were incubated in individual growth medium supplemented with 50, 25, or 12.5 mg/mL 10E8VLS or VRC01LS with or without 20% complement (V/V, Quidel) at 37°C for 4.5, 8.5, 25, and 48 hours. Control cells were incubated in corresponding growth medium supplemented with equal volumes of the formulation buffer for either 10E8VLS or VRC01LS. At each time point, 200 µL of supernatant was taken from each well and kept at –20°C before use. The supernatant was diluted by 3-fold in corresponding culture medium. The cytokine levels were measured by using a MilliPlex kit (MilliporeSigma, HADCYMAG-61K) on a Bio-Plex MAGPIX multiplex reader (Bio-Rad) following manufacturer’s instructions.

### Antibody aggregation in presence of serum

10E8VLS and VRC01LS stock solutions were diluted with PBS to obtain a 2-fold dilution series starting at 50 mg/mL up to 0.4 mg/mL in a 96-well 2 mL deep well plate. In total, 50 µL of each dilution was then added to individual wells of a clear 96-well plate containing 50 µL of RPMI 1640 media. FBS or normal HCS were then added to the wells at a final concentration of either 10% or 50% by volume. Immediately after the addition of FBS or normal HCS at room temperature, the optical density at 600 nm for each well was then measured using the SpectraMax Plus 384 Microplate Reader (Molecular Devices).

### Evaluation of complement activation

10E8VLS and VRC01LS stock solutions were diluted with PBS to obtain a concentration of 5 mg/mL, and 160 µL of each solution was added to separate 1.5 mL microcentrifuge tubes. Normal HCS was added at a volume of 40 µL to each condition/tube for a final concentration of 20% by volume. The tubes were then incubated for 30 minutes at 37°C and then spun down at 9,000*g* at room temperature for 5 minutes. The supernatant was then harvested and assayed for presence of various complement components (C1q, C3, C3b/iC3b, C4, and Factor B) using the MILLIPLEX MAP Human Complement Panel 2 — Immunology Multiplex Assay kit (MilliporeSigma) following manufacturer protocol. The plate was then read in the Flexmap 3D instrument (Luminex Corp) to obtain the levels of the various complement components for each condition tested.

### Activation of monocytes

PBMCs were isolated from buffy coats obtained from healthy blood donors from the NIH blood bank. Monocytes were enriched from PBMCs by negative selection using magnetic beads (Miltenyi Biotec), and monocyte purity was verified by flow cytometry after staining with antibodies against CD3, CD20, and CD14. Monocytes were then frozen and stored at liquid nitrogen. For the assay, the monocytes were thawed and kept overnight in RPMI culture media (Thermo Fisher Scientific) containing 0.1 mg/mL of IL-2, IL-4, and IL-7 along with 1% nonessential amino acids and 20 mM HEPES buffer. On day 0 of the assay, the monocytes were plated at a density of 100,000 cells per well of a 96-well culture plate, and test antibodies were added to the wells at a concentration of 100 µg/mL. After 3 days of culture, the supernatant was collected from each well and kept at –20°C before use. The supernatant was diluted by 3-fold in corresponding culture medium. The cytokines were measured using a MilliPlex kit (MilliporeSigma) on a Bio-Plex MAGPIX multiplex reader (Bio-Rad) following manufacturer instructions.

### Statistics

Group sizes (3–5 individuals) were calculated to capture SAEs and were comparable with other bNAb phase 1 trials. For a group size of 3 participants, there was a 90% chance of observing at least 1 SAE if the true event rate was more than 0.536 and a 90% chance of observing no SAE if the true event rate was less than 0.034. For a group size of 5 participants, there was a 90% chance of observing at least 1 SAE if the true event rate is more than 0.37 and a 90% chance of observing no SAE if the true event rate is less than 0.02. For comparison of binding to preadipocytes and adipocytes by 10E8VLS and VRC01LS and their secretion of IL-5, a 2-way ANOVA using the Šidák multiple-comparison test was performed. For comparison of serum aggregation and immune activation by 10E8VLS and VRC01LS, ordinary 1-way ANOVA with Tukey’s multiple-comparison test was performed. For these analyses, we considered any *P* value less than 0.05 as significant.

### Study approval

The clinical trial protocol was reviewed and approved by the NIAID IRB (Bethesda, MD). Patients were recruited using IRB-approved recruitment materials, and written informed consent was obtained from all participants prior to participation. Written informed consent was received for the use of the photographs and indicating that the record of informed consent has been retained.

### Data availability

Data generated in this study are available as deidentified data in ClinicalTrials.gov, NCT03565315, and in the Supporting Data Values file. The study protocol and informed consent form are available on ClinicalTrials.gov (https://clinicaltrials.gov/ProvidedDocs/15/NCT03565315/Prot_SAP_ICF_000.pdf). Additional data may be made available upon request from the corresponding author.

## Author contributions

GLC, CAC, MRG, JEL, EEC, NADR, RAK, JRM, ABM, SPH, RSSR, MCC, KC, CL, BCL, AP, SO, SK, YDK, PDK, FJA, and PJC conceptualized the study and designed the methodology. GLC, CSH, LAH, CAC, MRG, JEL, IJG, RD, OT, KC, and PJC performed the clinical investigation. AP, EEC, SFA, CGA, CL, LS, AMN, JPP, and EVC analyzed and visualized the trial data. JEL and JRM obtained funding for the study. MRG, JEL, IJG, RD, EEC, JRM, ABM, SPH, RSSR, OT, LS, MCC, KC, JGG, CL, FJA, and PJC performed project administration roles. GLC, CSH, LAH, CAC, MRG, JEL, IJG, RTB, EEC, RAK, SPH, CL, BCL, SRN, SO, FJA, and PJC performed supervisory roles. SFA, AP, CAC, MRG, and LS wrote the original draft of the manuscript. All authors critically reviewed and approved the final submission.

## Supplementary Material

Supplemental data

ICMJE disclosure forms

Supporting data values

## Figures and Tables

**Table 1:**
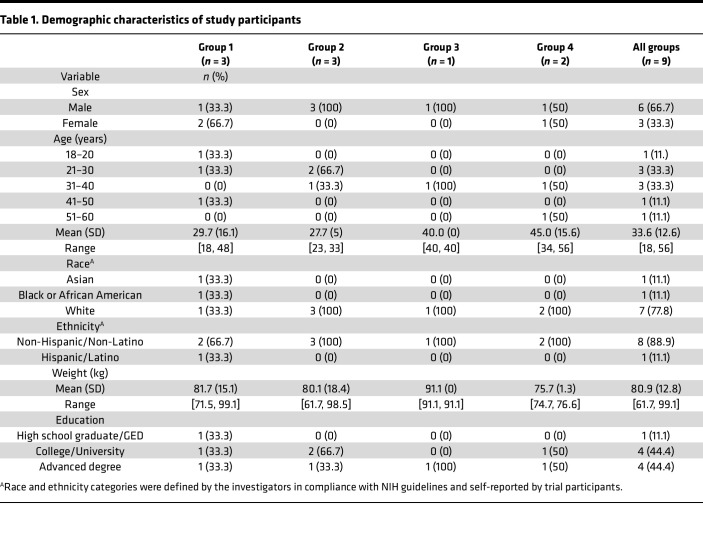
Demographic characteristics of study participants

## Version 1 04/08/2024

Electronic publication
