# Supplementary material for: Phase 1 trial evaluating safety and pharmacokinetics of HIV-1 broadly neutralizing mAbs 10E8VLS and VRC07-523LS
Source: JCI Insight. 2024 Apr 8;9(7):e175375. doi: 10.1172/jci.insight.175375 (PMC11128198; doi:10.1172/jci.insight.175375)
Supplement: Supplemental data [file jciinsight-9-175375-s225.pdf]

Supplementary Methods

10E8VLS and VRC07-523LS Serum Quantification:

The respective anti ID antibody (Anti-10E8VLS or 5C9 IgG2a; see ‘Generation of Target Antibody’ description below) solution was applied to the uncoated 96-well Meso Scale Discovery (MSD) plate (cat # L15XA-3) surface at a concentration of 1 mcg/mL for Anti-10E8VLS or 5 mcg/mL for 5C9. Plates were agitated for a brief time to ensure solution was fully coated over well bottom surface. Plates were sealed and placed at 4ºC overnight. The next day the plate was washed then blocked for 1 hour with a blocking solution (5% MSD Blocker A, cat # R93AA-1). An eight point, serially diluted, standard curve was prepared ranging in 10E8VLS concentration from 250 ng/mL to 3.9 ng/mL or VRC07-532LS concentration ranging from 150 ng/mL to 1.2 ng/mL. For 10E8VLS PK assay, the standard curve was prepared in 100-fold diluted Normal Human serum (NHS, cat #S1-100mL). Test samples were diluted 100-fold then serially diluted 2- or 3-fold through 7 points for a total of 8 dilutions on the plate. All samples and standards were run in duplicate. The blocking solution was washed, and reference or test samples were applied to the wells and allowed to incubate with shaking for one-hour Plates were washed to remove unbound sample. Sulfo-tag labeled anti human IgG detection antibody (Thermo Fisher cat # MH1015) was applied to the wells at 2 mcg/mL and allowed to associate with complexed Anti-10E8VLS: 10E8VLS or 5C9: VRC07-523LS within the assay wells. Plates were washed to remove unbound detection antibody. A read solution containing electrochemiluminescence substrate was applied to the wells, and the plate was entered into the MSD Sector instrument. A current was applied to the plate and areas of well surface which form a full Anti ID-target mAb-anti human IgG Sulfo-Tag complex emit light in the presence of the electrochemiluminescence substrate. The MSD Sector instrument quantitated the amount of light emitted and reported this electrochemiluminescence unit response as a result for each sample and standard of the plate. The amount of 10E8VLS or VRC07-523LS sandwiched by their respective Anti ID and Anti Human IgG antibodies is directly proportional to the concentration of reactive 10E8VLS or VRC07-523LS protein in the sample wells. Assuming the standard and test material are biologically similar, the responses generated from both materials can be compared to quantitate the concentration of 10E8VLS or VRC07-523LS in test samples. All calculations were performed within Excel and the GraphPad Prism software.

Generation of Target antibody:

Target antibody (VRC07-523LS or 10E8VLS) Fab was generated by LysC digestion and female Balb/c mice (Jackson Laboratory) were immunized i.m. twice with 20 mcg of antibody Fab in 100 mcL PBS and Ribi adjuvant (Millipore Sigma) at 0 and 4 weeks. Ten days post-boost, ELISA was used to test serum reactivity against target Fab and mice with high antibody reactivity were selected for cell fusion. Three days before cell fusion, antibody-reactive mice were boosted i.v. with 20 mcg of target Fab in 100 mcL PBS prior to spleen excision. Splenocytes were purified and fused with myeloma cells Sp2/0 (ATCC) in a 2:1 ratio according to established fusion protocols. Ten days post-fusion, triplicate hybridoma supernatants were screened for target antibody Fab reactivity. PCR was performed to amplify the heavy and light chain antibody genes of positive hybridomas, which were cloned into mouse IgG1 expression vectors (Gene Synthesis), expressed in Expi293 cells (Thermo Fisher Scientific), and purified over protein A (GE Healthcare). The best anti-idiotype clone was subcloned into a mouse IgG2a backbone and is referred to as Target antibody (VRC07-523LS or 10E8VLS) anti-idiotype (ID) antibody in all subsequent studies.

Supplementary Figures

Figure S1

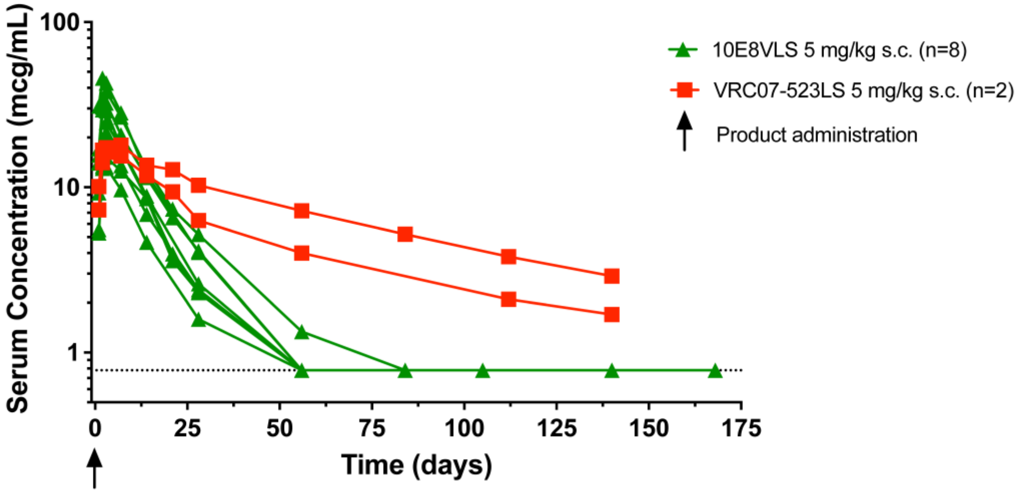

Figure S1. Individual antibody serum concentration (mcg/mL) of 10E8VLS and VRC07-523LS administered at 5 mg/kg s.c. All subjects who received 10E8VLS (n=8) or VRC07-523LS (n=2) are included. The black dotted line at y=0.78 indicates the limit of detection.

Figure S2

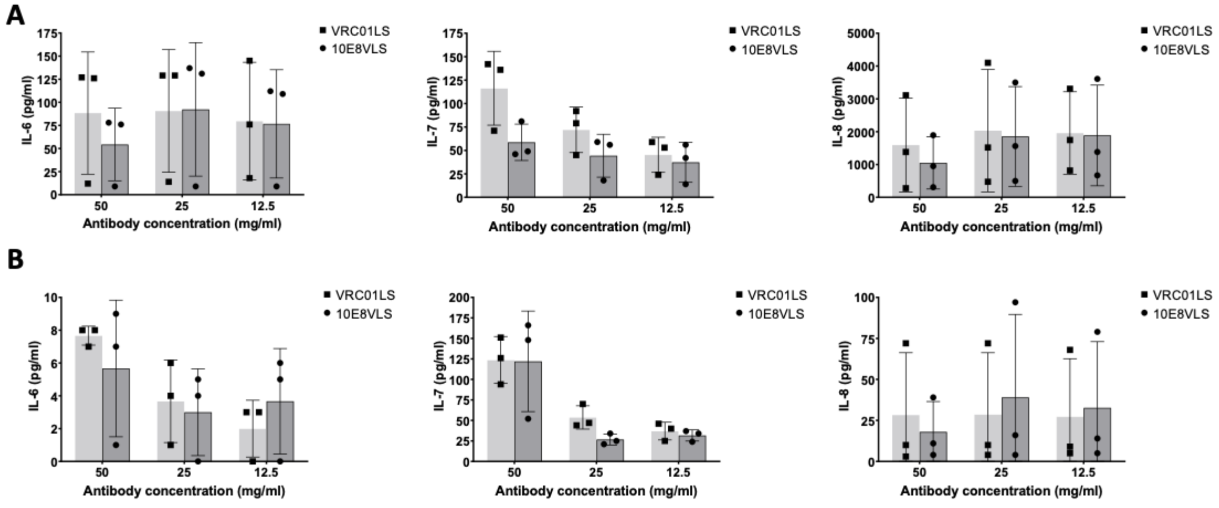

Figure S2. Secretion of IL-6, IL-7, and IL-8 by preadipocytes and adipocytes after incubation with 10E8VLS and VRC01LS. Primary human preadipocytes (A) and adipocytes (B) from three healthy donors were incubated with 50, 25, and 12.5 mg/mL 10E8VLS or VRC01LS. Culture supernatant was taken after a 4.5-hour incubation and assayed for secreted cytokine levels by a multiplex assay. The IL-6/7/8 levels in preadipocyte (A) and adipocyte (B) cultures are shown for each antibody concentration tested.

Figure S3

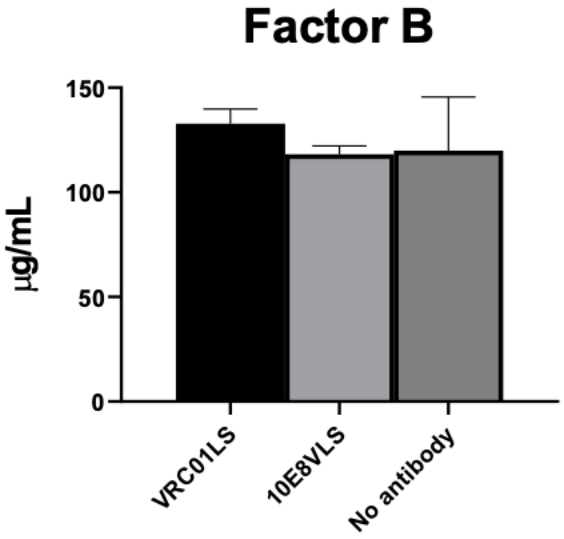

Figure S3. Effect on activation of complement protein Factor B by 10E8VLS or VRC01LS. 10E8VLS or VRC01LS at a concentration of 5 mg/mL, or PBS (no antibody) were incubated with 20% normal human complement serum. The levels of Factor B were measured after a 30-minute incubation by a multiplex assay.

Figure S4

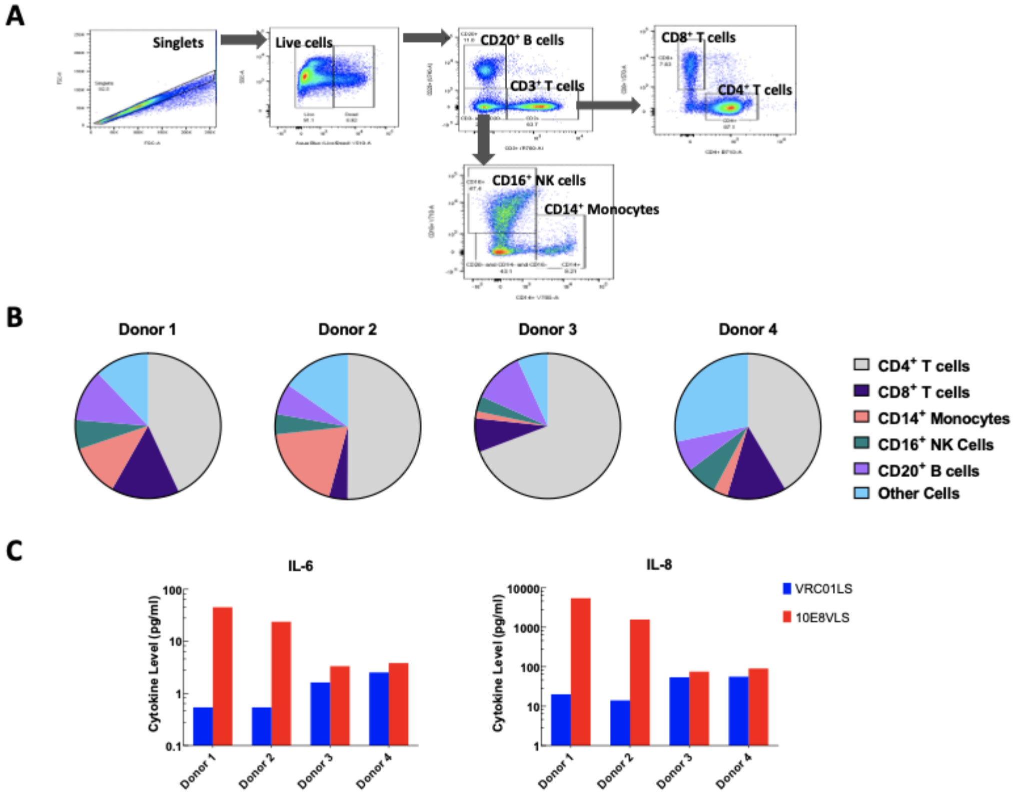

Figure S4. PBMC activation by 10E8VLS or VRC01LS. PBMCs derived from 4 healthy human donors were stained with a flow cytometry panel for assessing immune cell distribution and run on a flow cytometer. (A) A representative gating schema is showing the various immune cell subsets is shown. (B) The distribution of the immune cell subsets for each of the 4 donors is shown. (C) These PBMCs were then incubated with 100 mcg/mL of 10E8VLS or VRC01LS for 8 days and the levels of IL-6 and IL-8 in the culture supernatants were assessed at the end of the incubation period using a multiplex assay.

Figure S5

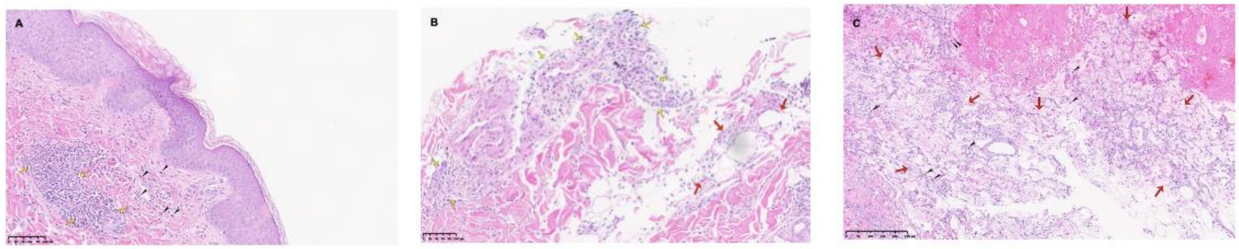

Figure S5. Right mid abdomen, skin biopsy. The Hematoxylin and Eosin (H&E) stains demonstrate both superficial (A) and deep (B, C) perivascular chronic inflammation with panniculitis and eosinophils. Immunohistochemistry of CD3 (not shown) labeled T-cells noted mainly in perivascular distribution. Black arrowheads highlight eosinophils. Yellow arrows outline areas with vascular inflammation. Red arrows outline areas demonstrating panniculitis.

Supplementary Tables

Table S1: Maximum local and systemic solicited reactogenicity

	5 mg/kg 10E8VLS Alone						5 mg/kg 10E8VLS + 5 mg/kg VRC07-523LS	
	**Participant 1**	**Participant 2**	**Participant 3**	**Participant 4**	**Participant 5**	**Participant 6**	**Participant 7**	**Participant 8**
**Volume Administered (mL)**	4.9 total<br>2.4 RLQ<br>2.5 LLQ	3.6 total<br>1.8 RLQ<br>1.8 LLQ	3.8 total<br>1.9 RLQ<br>1.9 LLQ	3.1 total<br>1.5 RUQ<br>1.6 LUQ	4.1 total<br>2.0 RLQ<br>2.1 LLQ	5.1 total<br>1.7 RLQ<br>1.7 RLQ<br>1.7 LLQ	10E8VLS sites:<br>4.7 total<br>2.3 RLQ<br>2.4 RUQ<br>VRC07-523LS sites:<br>4.7 total<br>2.3 LLQ<br>2.4 LUQ	10E8VLS sites:<br>3.7 total<br>1.8 LUQ<br>1.9 LLQ<br>VRC07-523LS sites:<br>3.7 total<br>1.8 RUQ<br>1.9 RLQ
**Solicited Local Reactogenicity**								
Pain/ Tenderness	Grade 1	Grade 1	Grade 1	Grade 1	Grade 1	Grade 1	10E8VLS: Grade 1<br>VRC07-523LS: None	10E8VLS: Grade 1<br>VRC07-523LS: None
Swelling	Grade 2 (7 cm) on days 0-4	None	Grade 2 (8 cm) on day 0	None	None	None	10E8VLS: Grade 2 (5 cm) on days 1-7<br>VRC07-523LS: None	10E8VLS: Grade 1 (4 cm) on days 1-5<br>VRC07-523LS: None
Redness	Grade 2 (7 cm) on day 1	None	Grade 2 (9 cm) on days 0-2	Grade 1 (4.6 cm) on days 0-4	Grade 2 (9 cm) on days 0-12	Grade 2 (7.8 cm) on days 1-5	10E8VLS: Grade 3 (18 cm) on days 0-70<br>VRC07-523LS: Grade 2 (8 cm) on days 1 and 10-45	10E8VLS: Grade 2 (7.6 cm) on days 0-9<br>VRC07-523LS: Grade 1 (3.5 cm) on days 0-2
Pruritis	None	None	None	Grade 1	None	None	None	None
Bruising	None	None	Grade 2	Grade 1	None	None	None	None
**Solicited Systemic Reactogenicity**								
Malaise	None	Grade 1 on day 1	None	None	None	None	Grade 1 on day 1	None
Nausea	None	Grade 1 on day 1	None	None	None	None	Grade 1 on day 1	None
Muscle Aches	None	None	None	Grade 1 on day 2	None	None	None	None
Chills	None	None	None	None	None	None	Grade 1 on day 1	None
Joint Pain	None	None	None	None	None	None	Grade 1 on day 1	None
Temperature	None	None	None	None	None	None	Grade 1 on day 1	None

RLQ – Abdominal right lower quadrant, RUQ – Abdominal right upper quadrant, LLQ – Abdominal left lower quadrant, LUQ- Abdominal left upper quadrant

Table S2: Maximum local and systemic solicited reactogenicity

	5 mg/kg 10E8VLS Alone *n = 6*	5 mg/kg 10E8VLS + 5 mg/kg VRC07-523LS *n = 2*	
**Local Symptoms**	***n (%)***		
	**10E8VLS Injection Site**	**10E8VLS Injection Site**	**VRC07-523LS Injection Site**
Bruising			
None	4 (66.7)	2 (100)	2 (100)
Mild	1 (16.7)	0 (0)	0 (0)
Moderate	1 (16.7)	0 (0)	0 (0)
Pain/ Tenderness			
None	0 (0)	0 (0)	0 (0)
Mild	100 (100)	2 (100)	2 (100)
Rash			
None	5 (83.3)	2 (100)	2 (100)
Mild	1 (16.7)	0 (0)	0 (0)
Redness			
None	1 (16.7)	0 (0)	0 (0)
Mild	1 (16.7)	0 (0)	1 (50.0)
Moderate	4 (66.7)	1 (50.0)	1 (50.0)
Severe	0 (0)	1 (50.0)	0 (0)
Swelling			
None	3 (50.0)	0 (0)	2 (100)
Mild	1 (16.7)	1 (50.0)	0 (0)
Moderate	2 (33.3)	1 (50.0)	0 (0)
Any			
None	0 (0)	0 (0)	0 (0)
Mild	2 (33.3)	0 (0)	1 (50.0)
Moderate	4 (66.7)	1 (50.0)	1 (50.0)
Severe	0 (0)	1 (50.0)	0 (0)
**Systemic Symptoms**	***n (%)***		
Arthralgia			
None	6 (100)	1 (50.0)	
Mild	0 (0)	1 (50.0)	
Chills			
None	6 (100)	1 (50.0)	
Mild	0 (0)	1 (50.0)	
Malaise			
None	5 (83.3)	1 (50.0)	
Mild	1 (16.7)	1 (50.0)	
Myalgia			
None	5 (83.3)	1 (50.0)	
Mild	1 (16.7)	1 (50.0)	
Nausea			
None	5 (83.3)	1 (50.0)	
Mild	1 (16.7)	1 (50.0)	

Temperature		
None	6 (100)	1 (50.0)
Mild	0 (0)	1 (50.0)
Any		
None	4 (66.7)	1 (50.0)
Mild	2 (33.3)	1 (50.0)

Table S3: Antidrug Antibody Analysis Results

		10E8VLS			VRC07-523LS		
**Subject**	**Time Point**	**Tier 1**	**Tier 2**	**Tier 3**	**Tier 1**	**Tier 2**	**Tier 3**
1	Pre-treatment	Negative	N/A	N/A			
	4 weeks post	Negative	N/A	N/A			
	8 weeks post	Positive	Negative	N/A			
2	Pre-treatment	Negative	N/A	N/A			
	4 weeks post	Negative	N/A	N/A			
	8 weeks post	Positive	Positive	Negative			
3	Pre-treatment	Positive	Positive	Negative			
	4 weeks post	Negative	N/A	N/A			
	8 weeks post	Negative	N/A	N/A			
4	Pre-treatment	Negative	N/A	N/A			
	4 weeks post	Negative	N/A	N/A			
	8 weeks post	Negative	N/A	N/A			
5	Pre-treatment	Negative	N/A	N/A			
	4 weeks post	Negative	N/A	N/A			
	8 weeks post	Positive	Negative	N/A			
6	Pre-treatment	Negative	N/A	N/A			
	4 weeks post	Negative	N/A	N/A			
	8 weeks post	Positive	Positive	Negative			
7	Pre-treatment	Negative	N/A	N/A	Negative	N/A	N/A
	4 weeks post	Negative	N/A	N/A	Negative	N/A	N/A
	8 weeks post	Negative	N/A	N/A	Negative	N/A	N/A
8	Pre-treatment	Negative	N/A	N/A	Negative	N/A	N/A
	4 weeks post	Negative	N/A	N/A	Negative	N/A	N/A
	8 weeks post	Negative	N/A	N/A	Negative	N/A	N/A

Tier 1: Standard Meso Scale Discovery (MSD) electrochemiluminescence detection
Tier 2: Competitive MSD electrochemiluminescence detection
Tier 3: Competitive HIV-1 pseudoneutralization assay

VRC 610 Study Team: The VRC 610 study team members not listed in the author line includes: Vaccine Research Center, NIAID, NIH: Joseph Casazza, Sarah Plummer, Allison Beck, Abidemi Ola, Alicia Widge, Jamie Saunders, Aba Eshun, Jennifer Cunningham, Brenda Larkin, Floreliz Mendoza, Laura Novik, Xiaolin Wang, William Whalen, Anita Arthur, Josephine H. Cox, Katherine V. Houser, Kaitlyn Morabito, Mario Roederer, Alison Taylor, Nina Berkowitz, Eugeania Burch, Maria Burgos Florez, Lam Le, Iris Pittman, Olga Vasilenko, Galina Yamshchikov, Martha Nason, Florence Kaltovich, Margarita Gomez Lorenzo, Sandra Sitar, Mark K. Louder, Stephen D. Schmidt, Kirsha McKee, Sijy O'Dell, Judy Stein, Christopher L. Case, Clare Whittaker, John Rathmann, Giune Padilla, and Christopher Moore; Vaccine Research Center Vaccine Production Program, NIAID, NIH: Lisa A. Kueltzo, Amy L. Chamberlain, Gabriel Moxey, Marianna Fleischman, Rahul Ragunathan, Rajoshi Chaudhuri, Ria T. Caringal, Slobodana D. Manceva, Vasha Hsu, Dan Gowetski, Paula Lei, Yile Li, Andrew Shaddeau, Nathan Barefoot, Erwin Rosales-Zavala, Yanhong Yang, Rong Yang, Xiangchun Eric Wang, Vera Ivleva, Nicole Schneck, Taryn Cozine, Janel Holland-Linn, Thomas Smith III, Elizabeth Scheideman, Rachel Kim, Nadia Amharref, Venkata Mangalampalli, Mingzhong Chen, Wei Cheng, and Lu Yang; and National Cancer Institute, NIH: Xueting Jin.
